# Cytogenetic and molecular identification of small-segment chromosome translocation lines from wheat-rye substitution lines to create wheat germplasm with beneficial traits

**DOI:** 10.1080/13102818.2014.901689

**Published:** 2014-04-29

**Authors:** Wei-Fu Song, Hai-Yan Ding, Xiao-Mei Zhang, Ji-Lin Li, Zhi-Min Xiao, Wen-Li Xin, Qing-Jie Song, Hai-Bin Zhao, Yan-Bin Zhang, Chun-Li Zhang

**Affiliations:** ^a^ Harbin Normal University, Biology Department, Harbin, P.R. China; ^b^ Daqing Normal University, School of Life Science, Daqing, Heilongjiang, P.R. China; ^c^ Heilongjiang Academy of Agricultural Sciences, Crop Breeding Institute, Harbin, P.R. China

**Keywords:** substitution line hybridization, genomic *in situ* hybridization, C-banding, wheat variety trials

## Abstract

Intergeneric crop plant hybrid lines with small-segment chromosome translocations are very useful in plant genetic research and breeding. In this study, to create small-segment chromosome translocations with beneficial agronomic characters, the progeny of wheat-rye substitution lines 5R/5A and 6R/6A were selected from generations F_2_ to F_5_ for rye-specific characteristics. A PCR primer and specific simple sequence repeat marker for rye were used in F_5_ populations to detect rye chromatin and to amplify a specific chromosome band in six translocation lines (06-6-5, 06-6-6, 06-6-9, 6-26-1, 7-23, and 7-33). Fragment pSc119.1 cloned from 7-33 had 99% homology with the big ear gene sequence (GenBank AF512607.1) in wheat. The six lines were further characterized via pollen mother cell meiosis analysis for genetic stability, and chromosome C-banding and genomic in situ hybridization for rye chromatin. The results show that line 7-33 was still within the 5R/5A substitution lines and possessed the big ear gene. The other lines all contained small-segment rye chromosome translocations. The results indicated that substitution line hybridization is an effective method for creating small-segment chromosome translocations with useful agronomic traits. Trials for these six wheat-rye translocation lines are justified because they possess many important stably-inherited agronomic characters, including disease resistance and improved yield.

## Abbreviations

BLAST: Basic Local Alignment Search Tool
C-banding: centromeric banding
gDNA: genomic DNA
GISH: genomic *in situ* hybridization
NCBI: National Center for Biotechnology Information
PCR: polymerase chain reaction
PMC: pollen mother cell
SSR: simple sequence repeat

## Introduction

The ability to transfer alien genes that are agronomically desirable from wild relatives into cultivated wheat is very important for the wheat geneticist and breeder (16, 24). Many wild relatives of common wheat (*Triticum aestivum* L.) possess genetic traits that, when incorporated into the wheat genome, have improved yield and resistance to various adverse environmental factors. Rye (*Secale cereale* L.) is closely related to wheat, and is a most useful source of alien genetic material for wheat-breeding. Several rye genes have already been successfully introgressed into the wheat genome, and these varieties are grown widely throughout the world for their disease resistance and other desirable agronomic traits (1, 2, 6, 10, 34).

Many studies have reported that through the creation of translocation lines, especially from alien chromosome fragments, alien genes or chromosome segments were transferred from wild relatives into common wheat (3, 12, 14, 22, 24). The best way to create translocation lines is by hybridization of wheat substitution lines (12). In our previous studies, we successfully obtained many translocation lines with good traits and heterozygous advantage by hybridizing the wheat-rye substitution lines 5R/5A and 6R/6A. However, the transferred chromosomal segments and genes in most translocation lines have not been identified.

To identify genes inherited from the rye parent, observation of phenotype can be the simplest and most direct method. For example, Sears (32) identified the 5R chromosome by its associated neck hair character, and Li et al. (15) identified the 4E chromosome from blue-grained wheat. In addition, many rye genome-specific repetitive DNA sequences are known and have been used to identify rye chromosomes or chromosomal segments in the wheat background. Examples of such sequences include pSc119.1, AF1/AF4, SCM120, SCM138, and SCM268 (17, 18, 26). Chromosome centromeric (C)-banding is another useful method for identifying chromosome translocations (36), and genomic *in situ* hybridization (GISH) is an efficient and accurate technique that detects all alien chromatin and the translocation breakpoints of interspecies chromosomal rearrangements (4, 11, 21, 24, 30) We, therefore, used pollen mother cell (PMC) meiosis analysis, chromosome C-banding and GISH to further identify the six lines.

In this study, we created translocation lines via hybridization of the wheat-rye 5R/5A and 6R/6A substitution lines, and then screened for rye-specific traits favorable for agronomy from hybrid generations F_2_ to F_5_. We identified those lines containing incorporated rye germplasm, using a specific molecular marker. In lines containing the amplified rye-specific fragment, stability of inheritance was assessed via PMC meiosis analysis. Rye chromatin was identified with chromosome C-banding and GISH.

## Materials and Methods

### Plant materials

The wheat-rye 5R/5A and 6R/6A substitution lines, described in detail in previous studies (12), were obtained from the Biology Department of Harbin Normal University. The lines 06-6-5, 06-6-6, 06-6-9, 6-26-1, 7-23, 7-33 are progeny of wheat-rye 5R/5A and 6R/6A substitution lines. The rye (2n = 14) was obtained from the Institute of Crop Germplasm Resources, Chinese Academy of Agricultural Sciences. Chinese Spring wheat (2n = 42) was provided by Kyoto University of Japan.

### Selection method

The maternal and paternal parents were crossed by conventional methods. All materials, including Chinese Spring wheat and rye, were grown in single-row plots 1.5 m long with 30 cm row spacing and 5 cm plant spacing within rows. Breeding for disease resistance against stem rust (*Puccinia graminis*) and powdery mildew (*Blumeria graminis*) was carried out in the greenhouse. The hybrid F_2_ to F_5_ generations were tagged for recording observations of plant height (cm), spike length (cm), number of spikelets per ear, number of grains per ear and grain yield per ear (g). All lines were selected according to phenotypic characters such as neck hair, abundant spikelets, disease resistance, and drought tolerance until stable (12).

### Cytogenetic and Molecular Identification of Small Chromosome Segment Translocation Lines

F_5_ generation lines with rye characters were assayed. Genomic DNA was extracted from seedlings, using cetyltrimethylammonium bromide (27). To amplify rye chromatin, two sets of the primer pair pSc119.1 (5′-TTGGCCCTCATGCCTTTAGTC-3′; 5′-CTTGGCCCTCTCCGCTTGACCG-3′) amplified a 750 bp fragment, and AF1/AF4 (5′-GGAGACATCATAAACATTT-3′; 5′-CTGTTGTTGGGCAGAAA-3′) amplified a 1500 bp fragment. Polymerase chain reaction (PCR) was performed using 1.0 U of Taq DNA polymerase (TaKaRa, DaLian, China) in 20 μL reaction volumes containing ∼50 ng of genomic DNA, 1× PCR buffer with 1.5 mmol/L MgCl_2_, 7.5 pmol of each PCR primer, and 100 μmol/L of each of the dNTPs. PCR cycling conditions for primers were 94 ºC for 5 min; and then 38 cycles of 94 ºC for 5 min, 94 ºC for 1 min, 55 ºC to 60 ºC for 1 min, 72 ºC for 30 s; and a final extension at 72 ºC for 10 min. Each PCR was repeated three times to preclude any technical errors.

All PCR products were resolved in a 1.4% agarose gel. The fragments were purified from the gels, and then cloned to a pGEM-T Easy vector (TransGen, China). Recombinant clones were sequenced after a PCR test. Sequencings were performed by Shanghai Sangon Biological Engineering Technology & Services (Shanghai, China). Sequence alignment was carried out with Basic Local Alignment Search Tool (BLAST) software. Six lines were assayed through simple sequence repeat (SSR) analysis. Primer sequences and chromosome locations are shown in (Table 1). PCR amplification was performed as described above. The examination and analysis of meiotic behavior and GISH was conducted as previously described by Li et al. (12), the latter with minor modifications. The procedures for C-banding and chromosome identification were in accordance with Gill et al. (7). Chromosome images were obtained with a charge coupled device (CCD) camera and analyzed with Photoshop (Version 8.0) software.

**Table 1.** SSR primer sequences and rye-specific chromosomal fragment verify the translocation line

Marker	Sequence	Product size (bp)	Repeat sequence	Chromosome arm
SCM120	F:CATTGTTGCGAGTGTTGAAGC	127	(AC)_10_	5RL
	R:TGTGCTGTCGTCGATGTTGTC			
SCM138	F:ATAGCCGCAGATGGTTGAGGAC	188	(AC)_23_	5RS
	R:GAGAAGTCTACAAATCAAGGGGGC			
SCM268	F:GCGCACCCCACACAACACG R:GCGGTGGCGGTTGAGGAC，	153	(CA)_9_	5RS

## Results and Discussion

The six lines have some beneficial traits and apparent rye characters, e.g., big ears, disease resistance, grey leaf, and heterozygous advantage (Table 2). In particular, line 7-23 has abundant spikelets and powdery mildew resistance; 7-33 has neck hair, a longer spike and abundant spikelets; 06-6-5 and 06-6-6 have abundant spikelets and delayed physiological maturity; 06-6-9 has abundant spikelets; 6-26-1 has abundant spikelets and the grain is shaped like that of rye. The six lines were judged to contain rye genetic material and utilized to screen for wheat-rye small-segment translocation lines. In the present study, the maternal wheat-rye substitution lines 5R/5A possess many obvious and useful traits, such as big ears, neck hair, purple coleoptiles, grey leaf, glumes, as well as drought tolerance and disease resistance. The paternal wheat-rye substitution lines 6R/6A has high spike density, strong glums, no arista, and is highly resistant to powdery mildew. Based on the characteristic traits of 5R/5A and 6R/6A, the plants were screened from the F2 to F5 generations. Observations of phenotype can indirectly identify the presence of chromosomes and genes from an alien genome in the wheat background (15, 32). The effect of alien chromosomal material was not consistent for wheat improvement (1, 33). Therefore, screening for yield components and disease resistance is the primary means of obtaining varieties of synthetically advanced character. In this study, the lines selected from the F5 showed many useful traits (**Table 2**). These results indicate that the desired target character can be obtained by screening the F_2_ to F_5_ generations.

**Table 2.** Morphological observations

							Disease resistance	
Lines	Morphological characters	Plant height (cm)	Spike length (cm)	Spikelets per ear	Grains per ear	Grain yield per ear (g)	Stem rust	Powdery mildew
7-23	No arista; purple coleoptiles; grey leaf;	77.2	13.6	26	52	1.6	R	HR
7-33	No arista; purple coleoptiles; neck hair	92.1	17.1	26	70	2.3	R	R
06-6-9	Long arista; grey leaf	85.3	14.6	26	65	2.1	R	R
06-6-6	Long arista; grey leaf; purple coleoptiles	97.2	15.48	27	51	1.9	R	HR
06-6-5	Long arista; grey leaf;	85.2	14.8	27	73	2.0	R	R
6-26-1	Long arista; grey leaf; glumes	98.5	13.74	20	40	1.2	R	R

R: resistance; HR: high resistance

**Figure 1.**
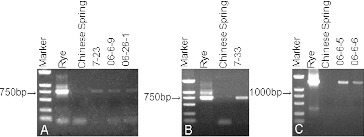
PCR amplification results. Amplification of pSc119.1 (**A** and **B**) and AF1/AF4 (**C**) in the translocation lines, rye, and Chinese Spring wheat. Marker DNA ladder 2000 (100 bp, 250 bp, 500 bp, 750 bp, 1000 bp, 2000 bp).

The precise identification of translocation lines is an important aspect in the evaluation and utilization of alien genetic material. Specific PCR markers are an easier and reliable means for detecting alien segments in the wheat background (28, 37). McIntyre et al. (18) was the first to report a molecular marker (Psc119.1) specific to and ubiquitous on a rye chromosome. Many molecular markers, covering almost the entire rye genome, have been developed in the last decades (17, 18, 26). Primer pSc119.1 amplified a 750 bp fragment as a rye-specific band in 7-23 (Figure 1A), 06-6-9 (Figure 1A), 6-26-1 (Figure 1A), and 7-33 (Figure 1B). Primer AF1/AF4 amplified a 1500 bp fragment as a rye-specific band in 06-6-5 and 06-6-6 (**Figure 1** **C**). The rye-specific PCR primers amplified specific bands in the rye and wheat-rye lines, whereas in Chinese Spring wheat neither of the rye-specific PCR primers could amplify any band. These results suggest that pSc119.1 and AF1/AF4 were rye-specific PCR primers, and the six lines were verified to contain rye chromatin. The PCR product of 7-33 was cloned and sequenced (**Figure 1** **B**). A search of GenBank (available through the National Center for Biotechnology Information [NCBI] website) and BLAST software showed that the DNA sequence of the analyzed fragment had 99.9% similarity to the big ear gene in wheat (GenBank accession number AF512607.1). This result suggests that 7-33 has the big ear gene.

**Figure 2.**
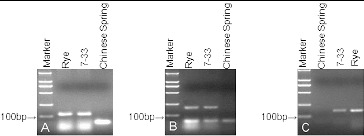
Agarose gel electrophoresis of the microsatellite markers SCM120 (**A**), SCM138 (**B**), and SCM268 (**C**) in lines 7-33, rye, and Chinese Spring wheat, indicating the presence of 5RL and 5RS. Marker DNA ladder 2000 (100 bp, 250 bp, 500 bp, 750 bp, 1000 bp, 2000 bp).

The SSR markers SCM120, SCM138, and SCM268 were used to identify 5R chromosomes (Table 1). SCM120 is located on rye 5RL and amplified a 127 bp fragment as a rye-specific band in 7-33 (**Figure 2** **A**). SCM138 and SCM268 are both located on rye 5RS and amplified a 188 bp and 153 bp fragment, respectively, as specific bands in 7-33 (**Figure 2** **B, C**). These results show that the translocation line 7-33 contained rye 5R chromosomes.

In the six translocation lines, meiotic univalents, heteromorphic bivalents, or multivalents were observed in metaphase. Chromosome bridges, chromosome fragments, and lagging chromosomes appeared in anaphase I and anaphase II. Micronuclei were found in the tetrad phase. The meiotic abnormal phenomena ratio was <2% in all phases of meiosis (**Table 3**), which indicated that heredity is stable in the six translocation lines.

**Table 3.** Observations and statistics of PMC meiosis

		Metaphase					Anaphase I				Anaphase II			
			UV (%)											
Lines	No. of cells	NB (%)	1–2	>2	HB (%)	MV (%)	LC (%)	CB (%)	CF (%)	LC (%)	CB (%)	CF (%)	MC (%)	TMN (%)
7-23	386	19.20	0.50	0.04 (3-5)	0.34 (1-4)	0.17 (3-5)	0.29 (1-3)	0.05 (1-4)	0.08 (1-2)	0.24 (1-3)	0.03 (1)	0.03 (1-2)	— —	0.24 (1-3)
7-33	382	19.87	0.36	0.01 (3-5)	0.43 (1-6)	0.16 (3-5)	0.63 (1-5)	0.03 (1-2)	0.09 (1-2)	0.22 (1-4)	0.06 (1-2)	0.02 (1)	0.01 (1)	0.30 (1-4)
06-6-9	399	20.10	0.11	0.02 (3-4)	0.44 (1-5)	0.24 (3-4)	0.05 (1-3)	0.18 (1-6)	0.06 (1)	0.01 (1)	0.05 (1-3)	0.01 (1)	—	0.01 (1-2)
06-6-6	375	20.70	0.07	—	0.22 (1-5)	—	0.08 (1-3)	0.07 (1-3)	0.11 (1-4)	0.06 (1-3)	0.03 (1)	—	0.01 (1)	0.09 (1-2)
06-6-5	368	20.09	0.23	0.07 (3-4)	0.57 (1-7)	0.01 (3)	0.20 (1-8)	0.10 (1-4)	—	0.20 (1-2)	0.09 (1-2)	0.02 (1)	—	0.21 (1-3)
6-26-1	359	20.11	0.08	0.01 (3-4)	0.51 (1-8)	0.08 (3-4)	0.35 (1-5)	0.28 (1-7)	0.13 (1-5)	0.13 (1-2)	0.07 (1)	0.07 (1-2	0.02 (1-2)	0.34 (1-7)

NB: normal bivalent; HB: heteromorphic bivalent; UV: univalent; MV: multivalent; LC: lagging chromosomes; CB: chromosome bridge; CF: chromosome fragment; MN: micronucleus; TMN: tetraspore micronucleus

**Figure 3.**
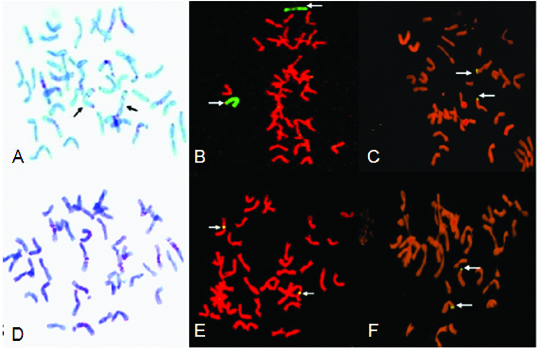
C-banding and GISH analysis of translocation chromosomes. C-banding pattern of lines 7-33 (**A**); arrows indicate two chromosomes with intense terminal C-band on arm. The chromosomes of 7-33 (**B**) after GISH using genomic DNA of rye as the probe; arrows indicate two substitution 5R chromosomes (green). The chromosomes of 06-6-9 (**C**) after GISH using genomic DNA of rye as the probe; arrows indicate small chromosome segment translocation. C-banding pattern of 06-6-9 (**D**); no rye chromosome band patterns. The chromosomes of 06-6-6 (**E**) and 06-6-5 (**F**) after GISH using genomic DNA of rye as the probe; arrows indicate small chromosome segment translocation.

Compared with the standard C-banding patterns of rye and Chinese Spring wheat, the translocation line 7-33 was missing the 5A chromosome but increased the 5R chromosome (**Figure 3** **A**). GISH indicated that there were two hybridization signals specific for rye chromosomes (**Figure 3** **B**). Together with the results of molecular identification, line 7-33 was designated as a 5R/5A substitution line. The rest of the lines were determined to be small-segment chromosome translocation lines, because no chromosome band patterns changed in the C-banding and because of the presence of a rye-specific hybridization signal in GISH analysis (**Figure 3** **C–F**). However, it could not be determined which of the wheat chromosomes contain these small rye chromosome segments.

Rye has many useful genes for wheat breeding and some of them have been transferred to wheat (6, 10, 23). Creating wheat-rye substitutions as well as translocations is an effective way of transferring rye chromosome material into wheat. For example, the wheat-rye 1BL/1RS translocation is the most widespread alien resource in wheat breeding worldwide due to its contribution to multiple disease resistance and positive effect on yield (8, 20, 23, 29, 33). Unfortunately, the transfer of the 1RS has resulted in deleterious effects on wheat end-use quality (8). To minimize the transfer of deleterious genes, the most desirable method for the plant breeder is to induce small chromosome segment translocations (25).

Ren and Zhang (24) created wheat-rye small-segment translocation lines using monosomic addition lines. Davies et al. (5) reported that some translocation lines were produced via hybridization between two *T. aestivum*–*S. cereale* addition lines. Li et al. (13) obtained translocation lines from the progeny of wheat–*Haynaldia villosa* and wheat–*Thinopyrum intermedium* disomic substitution lines and determined that the translocation lines were produced through exchange due to pairing of homoeologous chromosomes. Li et al. (12) created translocation lines through hybridization of wheat-rye substitution lines.

To create wheat-rye translocation lines possessing superior agronomical characteristics, in the present study, 5R/5A was crossed with 6R/6A. Via a tracing screen of target characteristics and cytogenetic and molecular identification, five chromosome segment translocations and one substitution were obtained in the F_5_ generation. This suggests that the crossing of substitution lines can create small-segment chromosome translocation lines with agronomic characteristics of polymeric quality.

The present study began with the crossing of the substitution lines 5R/5A and 6R/6A, and in the field the progeny from generations F_2_ to F_5_ were screened for rye characters. This ensured that traits in the translocation lines that had been selected for superior agronomic performance, such as high yield and resistance to stem rust and powdery mildew, were stable in the F_5_ generation. To avoid painstaking cytological work, we firstly utilized a rye-specific PCR primer to screen the F_5_ population for desirable rye characters. We found that specific PCR markers, pSc119.1 and AF1/AF4, amplified rye-specific bands in six lines and rye. However, no amplification products were found in Chinese Spring wheat. When amplification products of specific PCR markers for translocation were cloned and sequenced, we found the lines 7-33 to possess the big ear gene. These results document that the 5R or 6R chromosome of rye may carry the big ear gene; the definite location of this gene is still to be determined. The lines were then analyzed for genetic stability through observation of PMC meiosis. Identification of alien chromosomes was made using chromosome C-banding and GISH. This identification procedure enhanced accuracy and reliability, decreased the workload and saved time, and thereby gave the study a clear direction and focus.

The development of small-segment chromosome wheat-rye translocation lines that have beneficial agronomic traits has seldom been achieved (9, 19, 31, 35) although plant breeders have obtained them by several methods. Their creation via selection and then tracing target rye characters through generational progeny can exclude unwanted genes while sufficiently displaying the desired characters. Thus, small-segment chromosome translocation lines have great practical value in terms of improved yield and disease resistance in wheat.

## Conclusions

In this study wheat-rye chromosome translocation lines were created by crossing substitution lines. Through tracing selection, the translocation lines we produced have superior agronomic characters and improved wheat germplasm. In addition, these lines are useful for studying the mechanisms of alien chromosome introgressions into the receptor chromosome, and the expression of alien segments in receptor plants. Moreover, small-segment chromosome translocation lines can be used to study the mechanisms of gene exchange and gene expansion in species evolution.
